# Protocol for auxin-inducible depletion of the RNA-binding protein PTBP1 in mouse embryonic stem cells

**DOI:** 10.1016/j.xpro.2023.102644

**Published:** 2023-10-19

**Authors:** Yaroslav Kainov, Anna Zhuravskaya, Eugene V. Makeyev

**Affiliations:** 1 Centre for Developmental Neurobiology, King’s College London, London SE1 1UL, UK

**Keywords:** Cell Culture, CRISPR, Proteomics

## Abstract

Inducible degradation of proteins of interest provides a powerful approach for functional studies. Here, we present a protocol for tightly controlled depletion of the RNA-binding protein PTBP1 in mouse embryonic stem cells (ESCs). We describe steps for establishing an ESC line expressing doxycycline-inducible auxin receptor protein OsTIR1 and tagging endogenous *Ptbp1* alleles using CRISPR-Cas9 and homology-directed repair reagents. We then detail procedures for assaying the efficiency of inducible PTBP1 knockdown by immunoblotting. This protocol is adaptable for other protein targets.

For complete details on the use and execution of this protocol, please refer to Iannone et al.^1^

## Highlights

• Efficient knock-in of a Dox-inducible *OsTIR1* gene into mouse embryonic stem cell genome
• Protocol for tagging both alleles of a gene of interest with auxin-inducible degron sequences
• Recommendations for genotyping recombinant clones and assaying tagged protein degradation

## Abstract (editor highlights)

Publisher’s note: Undertaking any experimental protocol requires adherence to local institutional guidelines for laboratory safety and ethics.

## Graphical abstract

**Figure:**
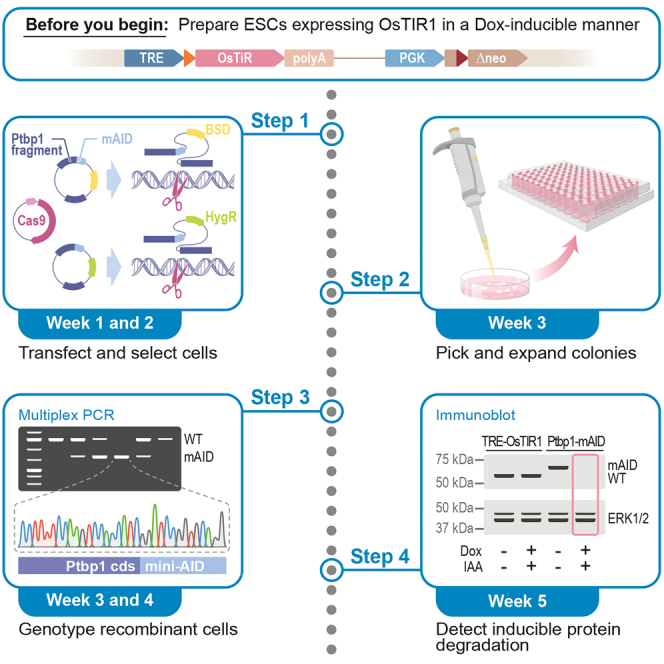

## Before you begin

The auxin-inducible degron (AID) system provides an efficient approach for rapid depletion of a protein of interest in mammalian cells.^2^^,^^3^ The system relies on a plant-derived ubiquitin ligase component that marks degron peptide-tagged proteins for proteosome-dependent degradation in the presence but not in the absence of the plant hormone auxin. The ability of this method to deplete specific proteins within just a few hours makes it a valuable tool for functional studies. For example, rapid depletion of an RNA-binding protein streamlines the analysis of its direct targets while minimizing any indirect effects on the transcriptome.

**Figure 1:**
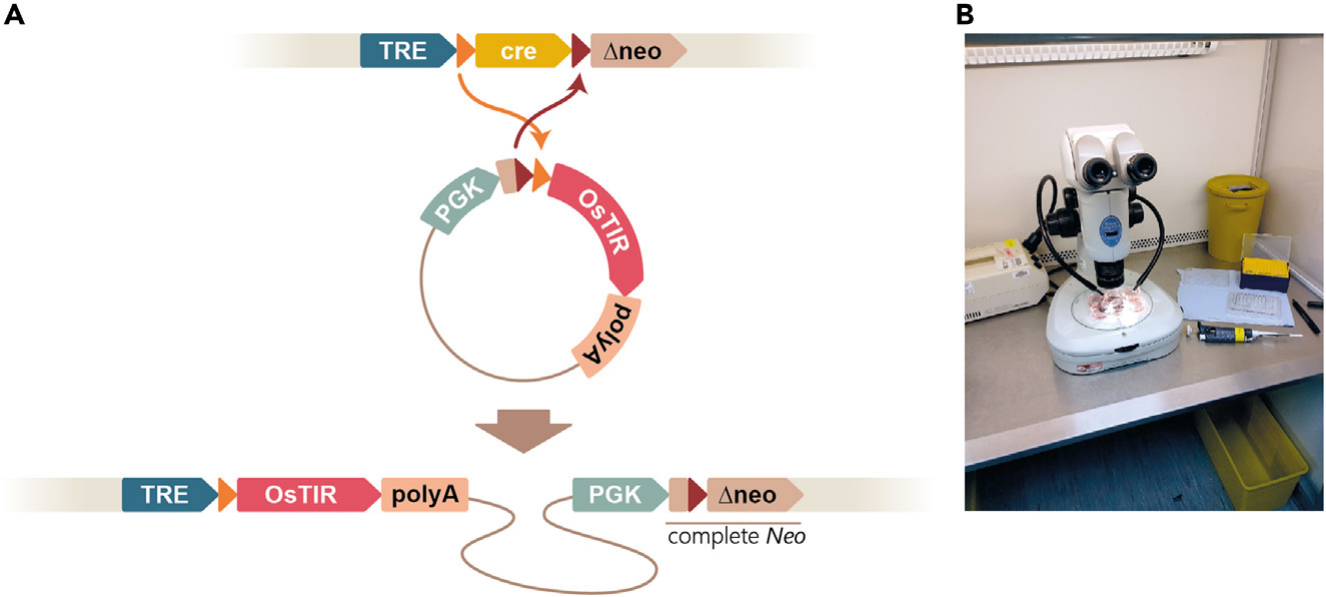
Preparing TRE-OsTIR1 ESCs (A) Cre/Lox recombination-mediated monoallelic knock-in of the *OsTIR1* transgene into the A2lox mouse ESC line. See^4^ for a more detailed description of A2lox system. (B) The setup we use to pick ESC colonies.

**Figure 2:**
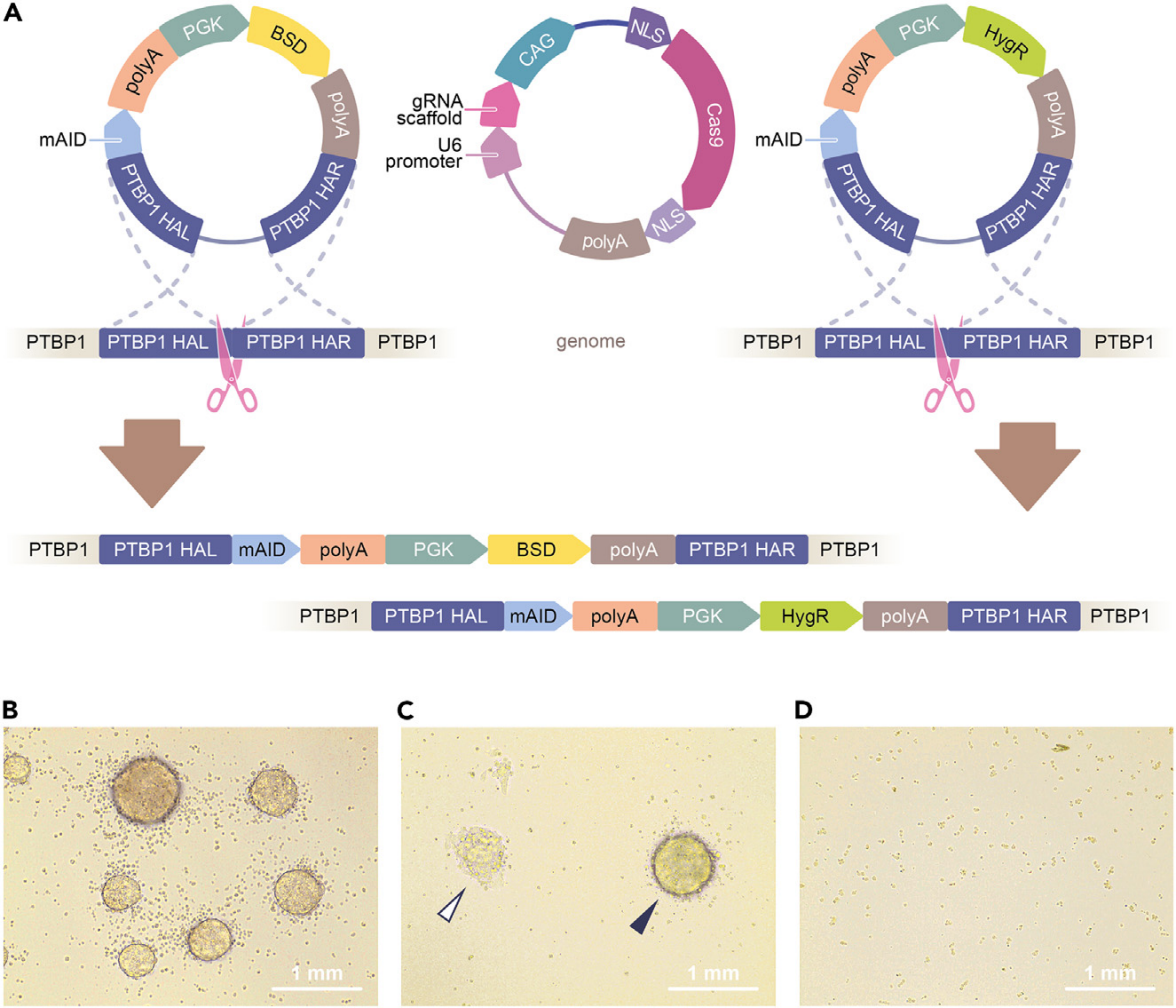
Biallelic tagging of the *Ptbp1* gene with *mAID* sequences (A) To knock in *mAID* into both allelic *Ptbp1* loci, TRE-OsTIR1 ESCs are co-transfected with an appropriate CRISPR-Cas9 plasmid and two homology-directed repair (HDR) constructs encoding the *HygR* and *BSD* selection markers. *Ptbp1 HAL* and *Ptbp1 HAR* and the left and right homology arms, respectively. (B–D) Examples of antibiotic-resistant ESC colonies formed in the experiment depicted in (A). (B) Undifferentiated colonies resistant to both hygromycin B and blasticidin S formed in a successfully co-transfected TRE-OsTIR1 ESC culture. (C) Another field containing an undifferentiated (filled arrowhead) colony suitable for picking and a differentiated colony (open arrowhead) that should not be used for further experiments. (D) No antibiotic-resistant colonies are detectable in a mock-transfected TRE-OsTIR1 ESC culture. Scale bars in (B-D), 1 mm.

**Figure 3:**
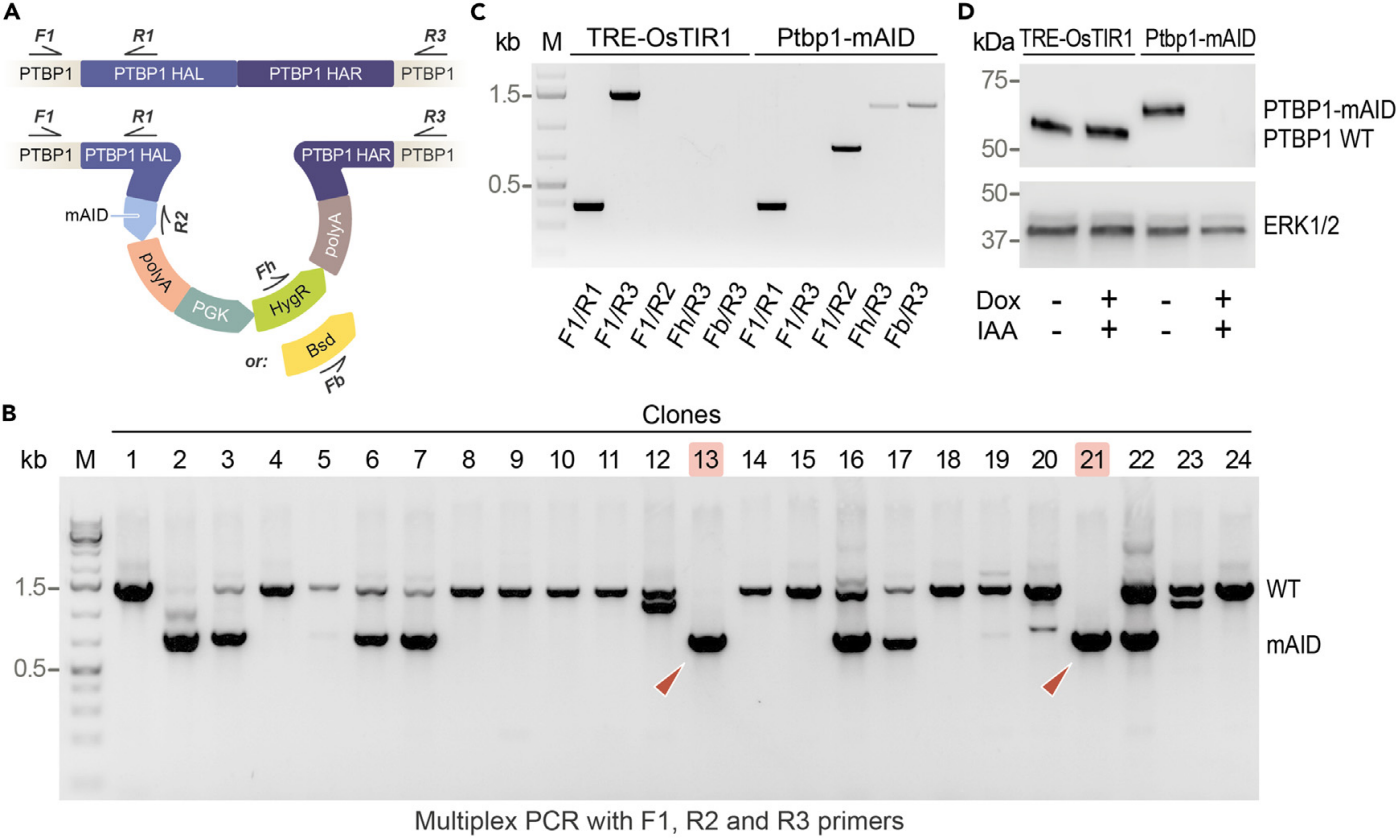
Genotyping *mAID* knock-in ESC clones and assaying inducible degradation of mAID-tagged PTBP1 (A) PCR genotyping strategy used to analyze *mAID* knock-in results. Top, the wild-type *Ptbp1* locus. Bottom, successfully targeted *Ptbp1* locus. See Table 1 for primer sequences. (B) Initial PCR screening of hygromycin B- and blasticidin S-resistant clones with primers introduced in (A). Arrowheads, clones 13 and 21 give rise to the mAID-specific but not the wild type-specific PCR product (expected product sizes are 775 bp and 1507 bp, respectively) indicating successful tagging of both *Ptbp1* alleles. (C) More detailed genotyping of the parental TRE-OsTIR1 ESC line and one of its Ptbp1-mAID clones with *Ptbp1* biallelically tagged with *mAID* using primer pairs indicated. The expected product sizes are: F1/R1, 385 bp; F1/R3, 1507 bp (wild type) and 3237 bp (mAID cassette knock-in; not detectable under the PCR conditions used); F1/R2, 775 bp; Fh/R3, 1298 bp; and Fb/R3, 1325 bp. (D) Immunoblot analysis showing stable expression of the wild-type PTBP1 in the parental TRE-OsTIR1 cells treated with Dox and IAA for 24 h. Conversely, and mAID-tagged PTBP1 is efficiently depleted in response to the Dox and IAA treatment in a biallelically tagged Ptbp1-mAID clone. As expected, mAID-tagged PTBP1 protein migrates slower than its wild-type counterpart (64.7 kDa vs. 56.9 kDa, respectively).

Here we present an optimized method for inducible depletion of the RNA-binding protein PTBP1 in mouse embryonic stem cells (ESCs). We first describe the procedures used to prepare media for feeder-free ESC cultures, propagate the parental ESC line A2lox, and retrofit it with a doxycycline-inducible transgene encoding the auxin-inducible ubiquitin ligase component OsTIR1 (Figure 1). We then provide a step-by-step protocol for CRISPR-Cas9 and homology-directed repair (HDR) mediated knock-in of *mini-AID* (*mAID*) sequences into the endogenous *Ptbp1* loci (Figure 2), as well as validating the knock-in clones and assaying them for inducible PTBP1 depletion (Figure 3). Although our description is focused on the PTBP1 example, we believe that the protocol can be readily adapted for other proteins of interest by selecting appropriate CRISPR-Cas9 and HDR constructs.

### Propagating ESCs in serum-free 2i+LIF medium

**Timing: 2–3 days per passage**

This part of the protocol describes routine cultivation of A2lox mouse embryonic stem cells, adapted from Mulas et al.^5^

**CRITICAL:** All cell media and solutions should be pre-warmed prior to use.

1. Defrost a vial of A2lox cells (a mouse ESC line for Cre-mediated knock-in of doxycycline-inducible transgenes^4^ ) at 37°C in a water bath. Immediately mix the cell suspension with 1–2 mL of pre-warmed DMEM/F12 medium and spin at 260 × *g* for 5 min, remove the medium and plate the cells in 5 mL of 2i+LIF to a 6-cm dish pre-coated with 0.1% gelatin (Merck, cat# ES-006-B). Grow the cells in a humidified incubator at 37 °C, 5% CO2. 70%–80% of cells should be attached 24 h after plating.

***Note:*** For coating, cover the bottom of a tissue culture (TC) grade 6-cm Nunc dish (Thermo Fisher Scientific, cat# 150318) with 2 mL of 0.1% gelatin and incubate the dish for 10–30 min at 37°C. Then aspirate gelatin, wash a dish with PBS and aspirate PBS prior to seeding cells.

2. Passage the cells every 2–3 days:
  a. Aspirate the medium, and rinse the plate with 2 mL PBS.
  b. Add 1 mL of 0.05% Trypsin-EDTA (Thermo Fisher Scientific, cat# 15400054 diluted 10 times with PBS), and incubate the plate for up to 10 min at 37*°*C.
    ***Note:*** Trypsinize cells at 37°C until ≥90% of the cells have detached. Observe the cells under the microscope to control detachment and avoid possible over-trypsinization.
  c. Quench trypsin with 4 mL of a 1:3 (v/v) mixture of ESC-grade FBS (Thermo Fisher Scientific, cat# SH30070.03E) and DMEM/F12 medium and pipette the suspension up and down a few times to dissociate cell clumps.
    ***Alternatives:*** To reduce costs, the concentration of FBS used for quenching can be decreased to 4%–5%.
  d. Collect the cells by centrifugation at 260 × *g* for 5 min, aspirate the supernatant, resuspend the pellet in 4 mL of Neurobasal medium and repeat the centrifugation.
  e. Resuspend the pellet in 2i+LIF and plate the cells to a fresh gelatin-coated dish at a 1:2–1:6 dilution.
    ***Note:*** ESCs tend to grow as dome-shaped, loosely attached colonies. Adding 1%–2% of FBS to 2i+LIF improves colony attachment.^6^ We occasionally use this approach to minimize colony loss in ESC cloning experiments (see below). However, FBS may also promote ESC differentiation and should be avoided during routine ESC passaging.
3. To cryopreserve A2lox cells:
  a. Remove the supernatant after the FBS quenching step and resuspend the cells in a required amount of the Recovery medium (Thermo Fisher Scientific, cat# 11560446).
  b. Prepare 0.5 mL aliquots in cryovials, place the aliquots into a Mr. Frosty freezing container (Thermo Fisher Scientific, cat# 5100-0001) at 20°C–25°C, and store the container at −80°C for 1–2 days.
  c. Transfer the vials to liquid nitrogen for long-term storage.

### Knocking in the OsTIR1 transgene into A2lox ESC genome

**Timing: 3–4 weeks**

This part of the protocol describes Cre-mediated knock-in of a single copy of the *OsTIR1* transgene into a *lox2272/loxP*-containing locus of the A2lox ES cell line developed in the Kyba lab.^4^

4. Subclone the open reading frame (ORF) of the auxin receptor F-box protein OsTIR1 into the p2Lox-GFP plasmid (Addgene #34635; Reference ^4^) in place of GFP using an appropriate cloning strategy.

***Note:*** We excised the OsTIR1 ORF from pMK232 (Addgene #72834; Reference ^7^) using the following three-step procedure: (1) NheI treatment, (2) filling in the NheI-generated ends with Klenow fragment and dNTPs, and (3) Acc65I treatment. The DNA fragment was then gel-purified and ligated with the vector fragment of p2Lox cut with EcoRV and BsrGI. This produced the pML33 (p2Lox-OsTIR1) plasmid (Addgene # 206996).

5. Passage A2lox cells 2–3 times after taking them from cryostorage, as described above.
6. On the day before transfection, add 1 μg/mL of doxycycline (Dox; Sigma, cat# D9891-1G) to the A2lox culture to activate the expression of the Cre recombinase for 16 h.

**CRITICAL:** This step is essential for successful knock-in of the *OsTIR1* transgene.

7. Next morning, trypsinize the culture, quench trypsin, and count the cells using hemocytometer. Spin down 0.75–1×10^6^ cells, discard the supernatant, and resuspend the pellet in 4 mL of 2i+LIF. Place the suspension into a 6-cm sterile bacterial dish (Corning, cat# BP53-06).

***Note:*** Bacterial 6-cm dishes are used to prevent cell attachment during transfection in suspension.

8. Transfect the cells by adding 1 μg of pML33 mixed with 3 μL of Lipofectamine 2000 (Thermo Fisher Scientific, cat# 11668019) and 100 μL of Opti-MEM I (Thermo Fisher Scientific, cat# 31985070) and incubating the suspension in a TC incubator for 2 h at 37°C (Figure 1A).

***Note:*** Use p2Lox-GFP and mock-transfected cells as a positive and negative control, respectively.

9. Collect the cells by centrifugation for 5 min at 260 × *g*, aspirate the supernatant, resuspend the pellet in 2 mL in 2i+LIF, and plate duplicated 100, 200 and 500 μL aliquots to individual wells of a gelatin-coated 6-well TC plate in the final volume of 2 mL of 2i+LIF per well.
10. After 24–48 h in a TC incubator:
  a. Replace the medium with 2 mL/well of 2i+LIF containing 350 μg/mL of G418/geneticin (Thermo Fisher Scientific, cat# 10131019).
  b. Propagate the cells for 10–15 days with regular medium changes (every 1–3 days depending on the extent of cell death) to allow G418-resistant cells to form colonies.

***Note:*** Colonies ready for picking should be visible without a microscope in cultures transfected with the pML33 and p2Lox-GFP plasmids but not in mock-transfected wells.

***Note:*** If colony detachment becomes a problem, add 1% of FBS to the medium 4–5 days prior to colony picking.

***Note:*** We recommend performing a killing curve assay to confirm the efficiency of G418 before beginning the cloning experiment.

11. Just before picking the colonies, pre-coat a flat-bottom 96-well TC-grade plate with gelatin as described above and add 200 μL of 2i+LIF with 2% FBS and 100 μg/mL of G418 per well. Also prepare a U-bottom 96-well plate (Greiner Bio-One, cat# 650180) with 50-μL aliquots of 0.05% Trypsin-EDTA per well.
12. Clean a stereomicroscope installed in a laminar flow cabinet with 70% ethanol (Figure 1B).
13. Find a well with well-separated G418-resistant colonies and dilute the conditioned medium in this well with 2 mL of PBS.
14. Pick the colonies using the stereomicroscope and a 20-μL automatic pipette equipped with sterile filter tips (Figure 1B). Gently dislodge a colony by pushing it with a tip, carefully suck it inside the tip, and deposit into a U-bottom well containing a 50-μL aliquot of 0.05% Trypsin-EDTA. Change the tip. Repeat.
15. Once all colonies are transferred to the U-bottom plate, incubate the plate at 37°C for 5–10 min.
16. Pipette the solution up and down with a multichannel pipette to dissociate the colonies and transfer the entire volume to the flat-bottom 96-well plate with 200 μL of 2i+LIF with 2% FBS and 350 μg/mL G418.
17. Next morning, change the medium to 2i+LIF with 100 μg/mL of G418 but without FBS.
18. When the cells in the 96-well plate reach 60%–70% confluence (typically in 2–4 days) trypsinize the cells and passage them through the 24-well plate, 6-well plate, and finally the 6-cm dish format.

***Note:*** In our experience, nearly all G418-resistant clones in the A2lox system express the transgene in a Dox-inducible manner. However, we recommend screening 6–12 clones and selecting the clones that grow well, form healthy-looking colonies, and express readily detectable amounts of OsTIR1 mRNA in Dox- but not control-treated samples (see below).

19. Cryopreserve the clones from 6-cm dish cultures using the procedure described in the "propagating ESCs in serum-free 2i+LIF medium" section. We typically prepare 6–8× 0.5-mL aliquots from a 60%–80% confluent 6-cm dish and use them to revive the cells in the 6-cm dish format.

**Pause point:** Clones can be cryopreserved as 1–2 aliquots at the 24-well or the 6-well passaging step and expanded when convenient.

20. To analyze OsTIR1 expression in the resultant TRE-OsTIR1 clones (*TRE* refers to the tetracycline/doxycycline inducible promoter driving *OsTIR1* expression in this system):
  a. Plate 0.5–1×10^5^ cells per well of a 12-well plate in 1 mL of 2i+LIF supplemented with 1 μg/mL of Dox.
  b. After at least 12 h, purify total RNA using the EZ-10 DNAaway RNA Miniprep Kit as recommended by the manufacturer.
    ***Alternatives:*** Other RNA purification methods can be used as an alternative to the EZ-10 DNAaway RNA Miniprep Kit. For example, we have a positive experience with Monarch Total RNA Miniprep Kit (NEB, cat# T2010S) and PureLink RNA Mini Kit (Thermo Fisher Scientific, cat# 12183018A).
  c. Generate cDNA using a reverse transcriptase (RT) kit of your choice (we use the SuperScript IV kit with random decamer primers as recommended by the manufacturer).
  d. Perform qPCR with OsTIR1_F/OsTIR1_R primers. Use *Cnot4* as a housekeeping control for qPCR signal normalization. See Table 1 for primer sequences.
    ***Note:*** We use the following amplification conditions:

**Table 1:** Oligonucleotides used in this protocol

Name	ID	Sequence, 5′ to 3′	Purpose
Ptbp1_gRNA_F	MLO697	CACCGCCCACAGGCACCTAGATGG	*Ptbp1*-specific gRNA cloning
Ptbp1_gRNA_R	MLO698	AAACCCATCTAGGTGCCTGTGGGC	*Ptbp1*-specific gRNA cloning
Ptbp1_HAL_F	MLO723	GGAAGTGAGCTCGCATTACACTGTCCAAGCA	Left homology arm cloning
Ptbp1_HAL_R	MLO724	ACAGGGGATCCGATGGTGGACTTGGAAAAGGACA	Left homology arm cloning
Ptbp1_HAR_F	MLO725	CATCTTCGAACCTGTGGGCCTGCATCAG	Right homology arm cloning
Ptbp1_HAR_R	MLO726	TATAAATGCCTAGGATTCGGGTATTTGGTCAAGTGG	Right homology arm cloning
BSD_F	MLO4126	GTGAGGCTAGCCACCATGGCCAAGCCTTTGT	*BSD* marker cloning
BSD_R	MLO4127	GTTATAGGTACCGAGCTCGAATTGTGCTTAGCCCTC	*BSD* marker cloning
OsTIR1_F	MLO773	ACAGGCCTGAACCTGAGCTA	Analysis of *OsTIR1* transgene expression
OsTIR1_R	MLO774	GGGAACACTCTCAGCTCCTG	Analysis of *OsTIR1* transgene expression
Cnot4_F	MLO461	CGCCACCCCAACCCTATACCA	Normalization of *OsTIR1* transgene expression
Cnot4_R	MLO462	GCCGAATGCTGCTTGCCAGT	Normalization of *OsTIR1* transgene expression
F1	MLO789	GTCCTGCTGCTCATGGTTTC	*mAID* knock-in genotyping
R1	MLO2576	TGAGGTCGTCCTCTGACACA	*mAID* knock-in genotyping
R2	MLO785	ACCATCACGTTCTTCCGGTA	*mAID* knock-in genotyping
R3	MLO1716	CCACAGGAACAGGCTAGGAT	*mAID* knock-in genotyping
Fb	MLO4156	GCTGGCAACCTGACTTGTATC	*mAID* knock-in genotyping
Fh	MLO1717	TTTCGATGATGCAGCTTGGG	*mAID* knock-in genotyping

**Table:** qPCR reaction mixture, 20 μL

Reagent	Volume
cDNA template	2.5 μL
2× qPCRBIO SyGreen Mix	10 μL
Primers mix (5 μM each)	1 μL
ddH_2_O	6.5 μL

**Table:** qPCR cycling conditions

Steps	Temperature	Time	Cycles
Initial Denaturation	95°C	600 s	1
Denaturation	95°C	10 s	45 cycles
Annealing	60°C	10 s	
Extension	72°C	10 s (signal acquisition)	
Hold	4°C	Indefinite

***Note:*** Use parental A2lox and the TRE-OsTIR1 transgenic cells without Dox treatment as negative controls.

***Optional:*** Analyze the expression of the OsTIR1 protein by immunoblotting with anti-OsTIR1 antibodies (cat# PD048, MBL Life Science) as described.^1^

## Key resources table

**Table:** 

REAGENT or RESOURCE	SOURCE	IDENTIFIER
**Antibodies**		

Anti-ERK1/2 (1:5,000 dilution)	Cell Signaling Technology	Cat# 4695S; RRID: AB_390779
Anti-PTBP1 (1:1,000 dilution)	Abcam	Cat# ab133734; RRID: AB_2814646
Peroxidase-AffiniPure Goat Anti-Rabbit IgG (H + L) (1:10,000–25,000 dilution)	Jackson ImmunoResearch	111-035-045-JIR-1.5ml, RRID: AB_2337938

**Bacterial and virus strains**		

TOP10 *E. coli*	Thermo Fisher Scientific	Cat# C404010

**Chemicals, peptides, and recombinant proteins**		

Neurobasal medium	Thermo Fisher Scientific	Cat# 21103049
DMEM/F12	Merck	Cat# D6421
Opti-MEM I	Thermo Fisher Scientific	Cat# 31985070
Trypsin-EDTA	Thermo Fisher Scientific	Cat# 15400054
Penicillin-Streptomycin (10,000 U/mL)	Invitrogen	Cat# 15140122
EmbryoMax 0.1% gelatin solution	Merck	Cat# ES-006-B
PD03259010	Cambridge Bioscience	Cat# SM26-2
CHIR99021	Cambridge Bioscience	Cat# SM13-1
L-glutamine	Thermo Fisher Scientific	Cat# 25030024
β-mercaptoethanol	Merck	Cat# M3148
ESGRO LIF	Merck	Cat# ESG1107
B-27 supplement without vitamin A	Thermo Fisher Scientific	Cat# 12587010
ES-grade FBS	Thermo Fisher Scientific	Cat# SH30070.03E
Progesterone solution	Merck	Cat# P8783-1G
Putrescine	Merck	Cat# P5780-5G
Sodium selenite solution	Merck	Cat# S5261-10G
Apo-transferrin	Merck	Cat# T1147-100MG
Insulin	Merck	Cat# I0516-5ML
Recovery Cell Culture Freezing Medium	Thermo Fisher Scientific	Cat# 12648010
Doxycycline	Merck	Cat# D9891
Blasticidin S	Merck	Cat# 15205-25MG
Hygromycin B	Merck	Cat# 400053-20ML
G418/geneticin	Thermo Fisher Scientific	Cat# 10131019
Lipofectamine 2000 reagent	Thermo Fisher Scientific	Cat# 11668019
Alt-R HDR Enhancer V2	IDT	Cat# 10007910
RQ1 RNase-free DNase	Promega	Cat# M6101
TURBO DNase	Thermo Fisher Scientific	Cat# AM2238
RNase inhibitor, murine	New England Biolabs	Cat# M0314L
SuperScript IV reverse transcriptase	Thermo Fisher Scientific	Cat# 18090200
PCRBIO HS Taq mix red	PCR Biosystems	Cat# PB10.23-02
qPCRBIO SyGreen mix	PCR Biosystems	Cat# PB20.11-51
GeneRuler 1 kb Plus DNA ladder	Thermo Fisher Scientific	Cat# SM1331
RIPA lysis buffer system	Santa Cruz Biotechnology	Cat# sc-364162
Halt protease inhibitor cocktail	Thermo Fisher Scientific	Cat# 78429
4× Laemmli sample buffer	Bio-Rad	Cat# 1610747
Immobilon western chemiluminescent HRP substrate	Merck	Cat# WBKLS0100

**Critical commercial assays**		

PCRBIO Rapid Extract PCR Kit	PCR Biosystems	Cat# PB10.24-08
Pierce BCA Protein Assay Kit	Thermo Fisher Scientific	Cat# 23227
EZ-10 DNAaway RNA Miniprep Kit	Bio Basic	Cat# BS88136

**Experimental models: Cell lines**		

A2lox mouse embryonic stem cells	Iacovino et al.^4^	N/A
Ptbp1-mAID mouse embryonic stem cells	This study	N/A

**Experimental models: Organisms/strains**		

A2lox mouse embryonic stem cells, *Mus musculus*, 129/Ola strain, male	Iacovino et al.^4^	N/A

**Oligonucleotides**		

See Table 1 for more detail	See Table 1 for more detail	See Table 1 for more detail

**Recombinant DNA**		

p2Lox	Iacovino et al.^4^	Addgene #34635
pMK232	Natsume et al.^7^	Addgene #72834
pX330	Cong et al.^8^	Addgene #42230
pML33	Iannone et al.^1^	Figure 1A; Addgene #206996
pML45	Iannone et al.^1^	Figure 2A; Addgene #206995
pML58	Iannone et al.^1^	Figure 2A; Addgene #206997
pML60	Iannone et al.^1^	Figure 2A; Addgene #206998
pML646	This study	Figure 2A; Addgene #206999

**Other**		

TC-grade 6-cm Nunc dishes	Thermo Fisher Scientific	Cat# 150318
Gosselin Petri dish 60 mm	Corning	Cat# BP53-06
U-bottom 96-well plate	Greiner Bio-One	Cat# 650180
Mr. Frosty Freezing Container	Thermo Fisher Scientific	Cat# 5100-0001

## Materials and equipment

**Table:** 100× N2 supplement stock, 10 mL

Reagent	Volume	Final concentration
DMEM/F12 medium	7.19 mL	N/A
BSA (75 mg/mL)	666.7 μL	5 mg/mL
Progesterone (0.6 mg/mL)	33.3 μL	2 μg/mL
Putrescine (160 mg/mL)	100 μL	1.6 mg/mL
Sodium selenite solution (3 mM)	10 μL	3 μM
Apo-transferrin (100 mg/mL)	1 mL	10 mg/mL
Insulin (10 mg/mL)	1 mL	1 mg/mL

***Note:*** Prepare 100 mg/mL apo-transferrin by dissolving the contents of a 100 mg vial in 1 mL DMEM/F12 immediately before making 100× N2. Use the 100× N2 solution to wash the vial and improve apo-transferrin recovery. Make sure that apo-transferrin is completely dissolved before aliquoting 100× N2.

***Note:*** Mix 100× N2 well by gently pipetting the final mixture up and down, aliquot by 1 mL, and store at -80°C for up to one year.

**Table:** Serum-free 2i medium with LIF (2i+LIF), ∼200 mL

Reagent	Volume	Final concentration
DMEM/F12 medium	100 mL	N/A
Neurobasal medium	100 mL	N/A
N2 (100*×*, see above)	1 mL	0.5*×*
B27 (50*×*, no vitamin A)	2 mL	0.5*×*
PenStrep (100*×*)	2 mL	1*×*
PD03259010 (10 mM)	20 μL	1 μM
CHIR99021 (10 mM)	60 μL	3 μM
β-mercaptoethanol (100 mM)	200 μL	0.1 mM
L-glutamine (200 mM)	0.5 mL	0.5 mM
ESGRO LIF (1 × 10^7^ units/mL)	20 μL	1000 units/mL

***Note:*** The medium can be stored for up to one month at +4°C.

***Note:*** Use freshly prepared 100 mM β-mercaptoethanol. Dilute 100 μL of β-mercaptoethanol with 14.1 mL of TC-grade sterile distilled H_2_O. Sterilize through a 0.2-μm filter and store at 4°C for up to 1 month.

## Step-by-step method details

This protocol describes tagging of both alleles of the *Ptbp1* gene with C-terminal mini-AID (mAID) sequences in TRE-OsTIR1 ESCs introduced above. We further outline the procedure for assaying inducible degradation of mAID-tagged PTBP1. In short, we co-transfect cells with a *Ptbp1*-specific CRISPR-Cas9 construct and two *Ptbp1*-specific HDR constructs with different selection markers. We provide HDR constructs with three markers to choose from: *HygR*, *BSD* and *PuroR* (conferring mammalian cell resistance against hygromycin B, blasticidin S and puromycin, respectively; Addgene ## 206997, 206998, 206999). However, we tend to obtain more biallelically tagged TRE-OsTIR1 clones using the *HygR* and *BSD* combination and describe this selection approach below. Once the Ptbp1-mAID knock-in clones (with the *TRE-OsTIR1, Ptbp1*^*mAID/mAID*^ genotype) are established and validated, they are treated with Dox and auxin (indole-3-acetic acid, or IAA), and the expression of OsTIR1 and mAID-tagged PTBP1 (PTBP1-mAID) is assayed by RT-qPCR and immunoblotting.

### Biallelic knock-in of mAID sequences

**Timing: 3–4 weeks**

This part of the protocol describes biallelic tagging of the mouse *Ptbp1* gene with auxin-inducible degron sequences. The TRE-OsTIR1 cells used for this purpose are described in the before you begin section.

1. Generate a CRISPR-Cas9 construct targeting the junction between the open reading frame and the 3′UTR in your gene of interest using an appropriate gRNA design tool (e.g., https://www.benchling.com).

***Note:*** We provide a Ptbp1-specific CRISPR-Cas9 plasmid (pML45; Addgene #206995) prepared by inserting the gRNA-encoding Ptbp1_gRNA_F/ Ptbp1_gRNA_R oligonucleotide duplex (Table 1) into pX330^8^ cut with BbsI.

***Note:*** In the example described in this section, the mAID tag is added to the C-terminus of the protein of interest. The knock-in strategy should be modified accordingly if N-terminal tagging is required.

2. Generate HDR constructs for in-frame C-terminal mAID tagging of your gene of interest with 2 different markers.

***Note:*** We provide three such constructs for Ptbp1 encoding the HygR, BSD, and PuroR eukaryotic selection markers (Addgene ##206997, 206998, 206999) and recommend using the HygR and BSD combination for targeting the TRE-OsTIR1 cells. To build these constructs, Ptbp1-specific left and right homology arms were amplified from mouse genomic DNA using the Ptbp1_HAL_F/Ptbp1_HAL_R and Ptbp1_HAR_F/Ptbp1_HAR_R primer pairs (Table 1), respectively. The left and the right arms were then cloned into pML42 (a HygR-encoding vector) and pML49 (a PuroR-encoding vector) at the SacI-BamHI and the BstBI-AvrII sites, respectively, producing the pML58 and pML60 HDR constructs.^1^ To prepare the BSD-containing HDR construct pML646, pML58 was cut with NheI-Acc65I and HygR was replaced by a BSD-containing fragment amplified from pLenti6-LacZ (Invitrogen) using the BSD_F/BSD_R primer pair (Table 1). For best results, we recommend keeping the length of the homology arms >500 bp.

3. Grow TRE-OsTIR1 cells in 2i+LIF with 100 μg/mL of G418 as described in the "knocking in the OsTIR1 transgene into A2lox ESC genome" section.
4. On the day of transfection, trypsinize the culture, quench trypsin, and count the cells using hemocytometer.
5. Transfect 0.75–1×10^6^ cells resuspended in 4 mL of 2i+LIF with a mixture containing 500 ng each of a CRISPR-Cas9 construct (e.g., pML45) and *HygR*- and *BSD*-containing HDR constructs (e.g., pML58 and pML646), 3 μL of Lipofectamine 2000 (Thermo Fisher Scientific, cat# 11668019) and 100 μL of Opti-MEM I (Thermo Fisher Scientific, cat# 31985070) in 6-cm bacterial dishes (Corning, cat# BP53-06) for 2 h (Figure 2A).

***Note:*** Use mock-transfected cells as a negative control.

6. Collect the cells by centrifugation for 5 min at 200 × *g*.
7. Resuspend the pellet in 2 mL of 2i+LIF, and plate duplicated 100, 200 and 500 μL aliquots to individual wells of a gelatin-coated 6-well TC plate in the final volume of 2 mL of 2i+LIF supplemented with 0.5 μM Alt-R HDR Enhancer V2 (IDT, cat# 10007910).
8. After 24–48 h in a TC incubator, replace the medium with 2 mL/well of 2i+LIF containing 150 μg/mL of hygromycin B and 8 μg/mL of blasticidin S.
9. Propagate the cells for an additional 10–15 days with regular medium changes (every 1–3 days depending on the extent of cell death) to allow antibiotic-resistant cells to form colonies.

***Note:*** We recommend performing killing curve assays to confirm the efficiency of hygromycin B and blasticidin S before beginning the cloning experiment.

***Note:*** Although mAID knock-in TRE-OsTIR1 cells (e.g., Ptbp1-mAID) remain resistant to G418, we do not use this antibiotic when selecting hygromycin B and blasticidin S resistant clones to avoid excessive cell stress. G418 can be added to the medium at 100 μg/mL once the mAID knock-in line has been established.

10. Pick and dissociate the colonies as described in the "knocking in the OsTIR1 transgene into A2lox ESC genome" section. Transfer the entire volume to a gelatinized TC-grade flat-bottom 96-well plate containing 200 μL of 2i+LIF with 2% FBS and 150 μg/mL of hygromycin B and 8 μg/mL of blasticidin S per well.

***Note:*** Colonies ready for picking should be visible without a microscope in cultures transfected with the CRISPR-Cas9 and HDR plasmid mixture but not in the mock-transfected control (Figures 2B–2D).

***Note:*** If colony detachment becomes a problem, add 1% of FBS to the medium 4–5 days prior to colony picking.

11. Next morning, change the medium to 2i+LIF without FBS and with 100 μg/mL of hygromycin B and 5 μg/mL of blasticidin S per well.
12. When the wells reach 60%–70% confluence (typically in 2–4 days):
  a. Trypsinize the cells.
  b. Quench with 200 μL of 2i+LIF with 2% FBS.
  c. Transfer half of the volume to a fresh gelatinized TC-grade flat-bottom 96-well plate containing 100 μL of 2i+LIF with 2% FBS and 100 μg/mL of hygromycin B and 5 μg/mL of blasticidin S per well.

***Note:*** Use the remaining half of the volume for DNA purification and PCR genotyping as soon as possible (see below).

13. Based on the PCR genotyping results (see below), select correct clones and passage them through the 24-well plate, 6-well plate, and 6-cm dish steps in 2i+LIF without FBS and with 100 μg/mL of hygromycin B and 5 μg/mL of blasticidin S.
14. Cryopreserve the clones from 6-cm dish cultures using the procedure described in the "propagating ESCs in 2i medium with LIF" section.

***Note:*** We typically prepare 6–8 × 0.5-mL aliquots from a 60%–80% confluent 6-cm dish and use them to revive the cells in the 6-cm dish format.

**Pause point:** Clones can be cryopreserved as 1–2 aliquots at the 24-well or the 6-well passaging step and expanded when convenient.

### Genotyping knock-in ESCs and assaying inducible degradation of mAID-tagged protein

**Timing: 1 week**

This part of the protocol describes molecular characterization of the mAID-tagged ESC clones.

**CRITICAL:** Perform genotyping as soon as possible to reduce the workload associated with passaging of numerous clones.

15. Centrifuge the 96-well plate with remaining half of trypsinized cells from step 12 of the previous section using a 96-well plate rotor at 1500 × *g* for 10 min.

***Note:*** We typically centrifuge cells in the flat-bottom plates where they grew. However, to analyze <60% confluent cultures, cell suspensions should be transferred to 96-well PCR plates prior to centrifugation to avoid losing the pellets.

16. Remove the supernatants using a multichannel pipette and extract the DNA from the cell pellets using PCRBIO Rapid Extract PCR Kit, as recommended by the manufacturer.

***Alternatives:*** Resuspend the cell pellets in the smaller volume of the final PCRBIO Rapid Extract mix (down to 30–40 μL) and incubate the plate for 5 min at 75°C and then for 10 min at 95°C.

**Pause point:** The lysates can be used directly for PCR genotyping or stored at 4°C for up to 24 h or at −20°C for up to a week.

17. To check whether the mAID-containing sequences were correctly integrated at the targeted loci, genotype the DNA samples from the previous step by multiplex PCR designed to amplify both the wild-type and knock-in alleles.

***Note:*** When tagging Ptbp1 with the pML45, pML58, and pML646 plasmid mixture, we performed this assay using F1, R2, and R3 primers (Table 1; Figure 3A) and the DNA polymerase from the PCRBIO Rapid Extract PCR Kit using the following conditions:

**Table:** PCR reaction master mix, 20 μL

Reagent	Amount
Genomic DNA template	2.5 μL
2× PCRBIO HS Taq Mix Red	10 μL
Primers mix (5 μM each)	1 μL
ddH_2_O	6.5 μL

**Table:** PCR cycling conditions

Steps	Temperature	Time	Cycles
Initial Denaturation	95°C	120 s	1
Denaturation	95°C	20 s	35 cycles
Annealing	58°C	30 s	
Extension	72°C	60 s	
Final Extension	72°C	120 s	
Hold	4°C	Indefinite

18. Analyze PCR products by agarose gel electrophoresis (Figure 3B) and select clones with robust amplification of the knock-in specific fragment and lacking the endogenous wild-type specific product for expansion (step 13 of the previous section) and protein immunodetection assays (see below).

***Note:*** In theory, F1/R3 primers can be used to differentiate between the wild type and mAID-tagged loci. However, the expected size of the mAID-tagged product is > 3 kb which may make it difficult to amplify it in a robust manner.

***Optional:*** Knock-in clones can be further verified by additional PCR assays described in Figure 3C and Sanger sequencing (not shown).

19. To analyze the efficacy of inducible protein degradation:
  a. Plate 0.5–1×10^5^ cells from each expanded clone of interest per well of 12-well plate in 1 mL of 2i+LIF supplemented with 1 μg/mL of Dox and 0.5 mM indole-3-acetic acid (IAA) and grow for 24 h.
  b. Collect the cells and analyze levels of protein of interest by immunoblotting with antibodies against the mAID-tagged protein (for mAID-tagged PTBP1, we use 1:1000 dilution of the cat# ab133734 antibody from Abcam; Figure 3D).

***Note:*** Use TRE-OsTIR1 and mAID-tagged (e.g., Ptbp1-mAID) cells treated with DMSO, or with Dox or IAA only as negative controls. If cytotoxicity becomes an issue, the concentration of IAA can be optimized within the 0.1–0.5 mM range.^7^

***Optional:*** Analyze the expression of the OsTIR1 protein by immunoblotting with anti-OsTIR1 antibodies (cat# PD048, MBL Life Science) as described.^1^

***Alternatives:*** Grow the 12-well cultures with 1 μg/mL of Dox for 16–24 h to pre-induce OsTIR1 expression and then supplement the wells with 0.5 mM IAA to trigger mAID-tagged protein degradation. In our experience, 4–6 h of IAA treatment of Dox-preinduced cultures is sufficient to reduce PTBP-mAID protein abundance to <10% of its original level.

## Expected outcomes

Expect to obtain 2–3 biallelic mAID knock-in clones after screening 40 hygromycin B- and blasticidin S-resistant clones. In immunoblot analyses of samples treated with Dox and IAA for 24 h (Figure 3D), the mAID-tagged protein should be completely degraded in the presence of both Dox and IAA but not when either or both compounds are omitted. If OsTIR1-specific antibodies are included in the assay^1^ the band of OsTIR1 protein should be detectable in the presence but not in the absence of Dox. In immunoblot analyses of Dox-preinduced cultures, it is expected that IAA should noticeably reduce the abundance of the mAID-tagged protein within a few hours.

## Limitations

The above protocol is based on the original auxin-inducible protein destabilization system developed by the Kanemaki lab.^7^ While it works well for PTBP1, it is possible that other proteins of interest may undergo leaky degradation in the absence of auxin or require high doses of this inducer for complete knockdown. In such cases, it may be advantageous to turn to the recently published "bump-and-hole" AID2 approach^9^ or other systems for inducible protein degradation.^2^

## Troubleshooting

### Problem 1

Low number of cells surviving after defrosting a cryostock (propagating ESCs in serum-free 2i+LIF medium, step 1).

### Potential solution

Make sure that an appropriate procedure was used when freezing down the cells. In particular:

• Recovery medium (or a home-made substitute suitable for ESCs freezing) was not accidentally diluted by the culturing medium;
• A sufficient amount of isopropanol was added to the Mr. Frosty container used for cell freezing;
• Cells were stored in the Mr. Frosty container for 1–2 days at −80°C prior to being transferred to liquid nitrogen;
• Frozen cells were thawed in a 37°C bath and promptly washed to remove DMSO-containing freezing medium

### Problem 2

Poor attachment of cells after plating (knocking in the OsTIR1 transgene into A2lox ESC genome, step 5).

### Potential solution

A2lox cells normally attach within a few hours and should be checked for attachment the next morning after plating. One possible reason for detecting numerous floating cells is a carry-over of trypsin. This problem can be tackled by increasing the amount of FBS at the quenching stage, more careful washing of the trypsinized and quenched cell pellet with Neurobasal medium or adding 1%–2% of FBS to the plating media. The latter approach can also improve the attachment of large ESC colonies in cloning experiments. However, supplementing the medium with FBS should be avoided during routine ESC passaging to avoid cell differentiation.

### Problem 3

No colonies after antibiotic selection (knocking in the OsTIR1 transgene into A2lox ESC genome, step 10).

### Potential solution

Make sure that:

• The cells were pre-treated with Dox prior to transfection to induce the expression of Cre-recombinase;
• The transfection mixture contained correct amounts of all the components were combined in the correct order;
• The ratio between plasmid DNA and Lipofectamine 2000 is within the range recommended by the manufacturer.

### Problem 4

No OsTIR1 expression in TRE-OsTIR1 cells (knocking in the OsTIR1 transgene into A2lox ESC genome, step 20).

### Potential solution

Make sure that the cultures were treated with Dox for at least 24 h prior to assaying OsTIR1 expression. Analyze a few additional transgenic clones.

### Problem 5

No homozygous clones with biallelic mAID knock-in (genotyping knock-in ESCs and assaying inducible degradation of mAID-tagged protein, step 18).

### Potential solution

Pick more colonies for genotyping. If this fails, increase the pX330-gRNA plasmid/HDR donor plasmid ratio and repeat the experiment.

### Problem 6

Poor expression of mAID-tagged protein in uninduced cells (genotyping knock-in ESCs and assaying inducible degradation of mAID-tagged protein, step 19).

### Potential solution

Analyze additional clones to rule out clone-specific mutations. If the antibody used for immunodetection was raised against a C-terminal epitope, consider using a different antibody whose epitope is unlikely to be disrupted by the C-terminal mAID tag. If this fails, consider N-terminal mAID tagging or use a different inducible degradation system.^2^^,^^3^

## Resource availability

### Lead contact

Further information and requests for resources and reagents should be directed to and will be fulfilled by the lead contact, Eugene V. Makeyev (eugene.makeyev@kcl.ac.uk).

### Materials availability

All reagents used in this study are described in the key resources table. Plasmids are available from Addgene.

## Data Availability

No datasets or custom code were generated in this study.
